# Chronic pain and mortality risk among middle-age and older Japanese: the Shika town cohort study

**DOI:** 10.1097/PR9.0000000000001361

**Published:** 2025-11-12

**Authors:** Jam Camara, Hiromasa Tsujiguchi, Akinori Hara, Yoko Inagaki, Masaharu Nakamura, Chie Takazawa, Keita Suzuki, Sakae Miyagi, Fumihiko Suzuki, Tomoko Kasahara, Atsushi Asai, Koji Katano, Kuniko Sato, Aya Ogawa, Shinobu Fukushima, Hirohito Tsuboi, Yukari Shimizu, Aki Shibata, Yasuhiro Kambayashi, Takayuki Kannon, Jiaye Zhao, Talica Marama, Yumie Takeshita, Atsushi Tajima, Toshinari Takamura, Hiroyuki Nakamura

**Affiliations:** a Department of Public Health, Graduate School of Advanced Preventive Medical Sciences, Kanazawa University, Kanazawa, Ishikawa, Japan; b Department of Hygiene and Public Health, Faculty of Medicine, Institute of Medical, Pharmaceutical and Health Sciences, Kanazawa University, Kanazawa, Ishikawa, Japan; c Advanced Preventive Medical Sciences Research Center, Kanazawa University, Kanazawa, Ishikawa, Japan; d Department of International Health, Johns Hopkins Bloomberg School of Public Health, Baltimore, MD, USA; e Innovative Clinical Research Center, Kanazawa University, Kanazawa, Ishikawa, Japan; f Department of Geriatric Dentistry, Ohu University School of Dentistry, Koriyama, Fukushima, Japan; g Faculty of Nutrition, Osaka Seikei College, Higashiyodogawa-ku, Osaka, Japan; h Department of Public Health, Faculty of Veterinary Medicine, Okayama University of Science, Imabari, Ehime, Japan; i Department of Biomedical Data Science, School of Medicine, Fujita Health University, Toyoake, Aichi, Japan; j Department of Endocrinology and Metabolism, Graduate School of Medical Sciences, Kanazawa University, Kanazawa, Ishikawa, Japan; k Department of Bioinformatics and Genomics, Graduate School of Advanced Preventive Medical Sciences, Kanazawa University, Kanazawa, Ishikawa, Japan

**Keywords:** Chronic body pain, Longitudinal, Middle-aged and elderly, Mortality, Community dwellings

## Abstract

**Introduction:**

The relationship between chronic pain (CP) and mortality among the Japanese elderly population remains unclear due to limited literature.

**Objectives:**

This study aimed to investigate the relationship between CP and mortality, stratified by age and sex, using longitudinal cohort data from the Shika Study.

**Methods:**

This was a longitudinal study with 2849 participants aged ≥40 years (44.75% males and 55.25% females). Chronic pain, age, sex, tobacco smoking, height, and weight and self-reported information on medical conditions such as hypertension, hyperlipidemia, and diabetes mellitus were assessed using a CP questionnaire.

**Results:**

The baseline mean age (years) was 64.00 ± 12.21 for men and 65.44 ± 13.12 for women. Among all participants, the mortality rate was significantly higher in males than in females (12.40% vs 7.80%; *P* < 0.001). The mortality rates among those with chronic headache (alive = 1.40% vs dead = 9.10%; *P* = 0.004) and neck/shoulder/elbow/hand pain (alive = 15.50% vs dead = 31.80%; *P* = 0.038) were significantly higher among the dead group compared to those alive in women. The odds of mortality associated with chronic headache (9.238 [95% CI 1.729–49.352]; *P* = 0.009) and neck/shoulder/elbow/hand pain (adjusted odd ratio 2.586 [95% CI 1.012–6.608]; *P* = 0.047) were significantly higher in females aged ≤74 years but not in males.

**Conclusion:**

Chronic headache and neck/shoulder/elbow/hand pain were found to be independently associated with mortality irrespective of other covariates among Japanese rural women aged ≤74 years in the Shika town longitudinal cohort study, Japan. This study potentially highlights the public health importance and negative impact of CP in women aged ≤74 years.

## Abstract 2

Supplemental Digital Content is Available in the Text.

Chronic pain is independently associated with mortality irrespective of other covariates among Japanese rural women aged ≤74 years in the Shika town longitudinal cohort study, Japan.

## 1. Introduction

A systematic analysis of the 2019 World Health Organization (WHO) Global Burden of Disease Study revealed that 1.71 billion people were living with musculoskeletal conditions, including low back pain, neck pain, fractures, osteoarthritis, and rheumatoid arthritis.^10^ Musculoskeletal health encompasses the performance of the locomotor system, including muscles, bones, joints, and adjacent connective tissues.^25^ These conditions were the leading cause of disability, accounting for 149 million (17%) years lost to disability worldwide in 2019.^10^ Global Burden of Disease further highlighted that over half a billion people globally will live with chronic pain (CP) by 2030.

Systematic review studies have shown that CP is a leading cause of disability and a common reason adults seek care, with estimated annual costs of approximately $700 billion in the United States.^51,56^ In addition, higher body mass index (BMI) in US adults is associated with increased rates of CP.^44^ A study comparing health outcomes in the United States to those in 34 other Organization for Economic Co-operation and Development countries found that 3 of the 4 leading causes of year-life-lost were related to CP: back pain, musculoskeletal disorders, and neck pain. Notably, morbidity and chronic disability account for nearly 50% of the US health burdens.^55^ Similarly, studies have shown that older females have higher prevalence rates of rheumatoid arthritis and osteoporosis compared to males, as seen in the National Health and Nutrition Examination Survey (NHANES)^27^ and a systematic review with 204 countries using 98 prevalence and 25 incidence studies.^5^ Hence, CP and high-impact CP leads to excess mortality, compared with the absence of CP.^39^

Individuals with CP are more likely to be smokers, alcoholics, obese, inactive, and have comorbidities such as depression, anxiety, emotional problems, and sleep disturbances.^39^ These factors contribute to increased mortality outcomes among people with CP and high-impact CP compared to those without CP.^39^ Rahini et al.^36^ found that osteoarthritis accounts for 15% of all musculoskeletal disorders, with 69.2% of knee osteoarthritis cases occurring in female patients over 45 years old.

Numerous studies have shown that CP is highly prevalent among older adults, contributing to disability, social isolation, and increased health care burdens.^33,41,42^ Thus, CP is a risk factor for cognitive decline, suggesting potential shared mechanisms with dementia.^38,49^ The association between persistent pain and the risk of accelerated cognitive decline complicates the estimation of CP in older populations.^15^ Inflammatory processes in rheumatoid diseases, cardiovascular diseases, and CP are known to be associated with an increased risk of premature death.^11,14^ A Danish study found higher all-cause and cause-specific mortality among individuals with musculoskeletal pain compared to those without pain.^17^

A Japanese study found that CP was most prevalent in the neck, shoulder, and lower back among 18- to 50-year-olds, with a prevalence of 15.4%.^31^ In another Japanese study, authors identified risk factors for developing CP, including working in professional, managerial, or clerical occupations, being female, having a BMI ≥ 25 kg/m^2^, current alcohol or cigarette use, and having a higher education level (vocational school or above).^32^

The report on musculoskeletal diseases of 2022 in the WHO database showed an almost twofold higher mortality rate in Japanese females with CP than their male counterparts.^53^ The increased mortality rates among females with CP in Japan are concerning, as CP also leads to functional limitations and substantial burdens on health care and government expenditures. Therefore, clarifying the relationship between CP and mortality according to sex and age is crucial for addressing these issues.

There is limited evidence on the association between CP and mortality among Japanese middle-aged and older adults, and it remains unclear how much CP affects mortality by age and sex. Therefore, our study investigated this relationship among community-dwelling adults using the Shika Town Longitudinal Cohort Study in Ishikawa, Japan. We hypothesized that mortality among middle-aged and elderly Japanese individuals with CP is as a result of aging, high BMI, and alcohol intake.

## 2. Methodology

### 2.1. Participants and study area

The study design was longitudinal, with baseline data collected between 2011 and 2016 from Shika town in Ishikawa Prefecture, located on the Noto Peninsula in Japan. The principal industries in Shika town include electronic manufacturing, retail, medical care, and welfare, with agriculture and fishery being secondary industries, with a highly aging population of 31.19% (≥65 years) according to the Shika town population data in September 2011, providing a suitable demographic context for investigating age-related health outcomes.

**Figure 1.**
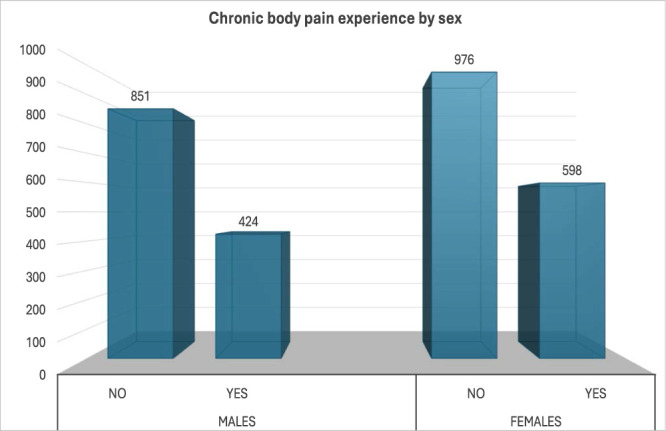
Showing participants with or without pain experience by sex.

Middle-aged and elderly individuals residing in the town of Shika, Japan, were recruited. All residents aged 40 years and older were listed with the help of the Shika Municipal government. Among the 5013 eligible residents, 4628 participated in this study (rate = 92.32%). After excluding 698 participants due to missing data (202 lacked sex data, 118 lacked pain location/intensity data, and 379 lacked smoking data), 2849 participants aged ≥40 years (1275 males and 1574 females) with complete datasets were included in this analysis(Fig. 1).

### 2.2. Research tools

A self-administered questionnaire containing questions about demographic information, such as age, sex, height, weight, and tobacco smoking, and self-reported information on medical conditions, such as hypertension, hyperlipidemia, diabetes mellitus, and CP locations and severity, was administered. Alcohol intake was assessed using the brief self-administered diet history questionnaire (BDHQ).^1^ Details of the BDHQ were described elsewhere.^41^ Mortality data with consent from the participants were obtained from the vital registrations of the Shika Municipal government until December 2023.

The average follow-up durations for all participants (8.64 ± 2.45 years), survivors (8.96 ± 2.19 years), and nonsurvivors (5.71 ± 2.76 years). When examined by sex, survivors were (male: 8.91 ± 2.16, female: 9.00 ± 2.21) and nonsurvivors (male: 5.55 ± 2.93, female: 5.91 ± 2.52), respectively. Chronic pain was defined as pain persisting for 3 months or longer based on the International Statistical Classification of Diseases and Related Health Problems (ICD)-11.^3,17^ Participants indicated the first, second, and third most painful locations on their body and rated CP severity on a scale of 0 (no pain) to 10 (most severe). A pain intensity score ≥1 indicated CP when it persisted for 3 months or more. The CP locations assessed included the head, neck, shoulder, elbow, hand, upper back, lower back, hip, knee, foot, chest, and stomach. These locations were categorized based on proximity and the relationship between pain sites, as well as the limited number of participants in each site: head,^1,4,23,29^ neck/shoulder/elbow/hand,^1,2^ knee/foot,^23^ and upper back/lower back/hip (the questionnaire is attached as supplementary material, http://links.lww.com/PR9/A358).

### 2.3. Statistical analysis

Data were summarized using descriptive statistics. Continuous variables were described as means and standard deviations (SD), and discrete variables were described as frequencies and proportions. The Student *t*-test and χ^2^ test were used to compare continuous and categorical variables, respectively. Logistic regression analysis was performed to examine the association between CP and mortality stratified by sex and age. In the logistic regression analysis, mortality (alive = 0, dead = 1) was the dependent variable, and CP (no pain = 0, having pain = 1) was the independent variable. All selected covariates were based on literature identifying known risk factors and proxies for mortality among individuals with chronic pain.

The logistic regression model was adjusted for age,^1,7,26,44^ BMI,^1,44,50,52,54^ alcohol intake,^1,16^ tobacco smoking,^1,7,8,17,19^ high blood pressure,^1,9,40^ diabetes,^1,7,9,40^ and hyperlipidemia.^1,9,40^ The age classification was based on the Joint Committee of Japan Gerontological Society and the Japan Geriatrics Society's proposal, as follows: ≤74 years and ≥75 years, applied to both male and female participants.^34^ All tests were 2-tailed, and differences were considered statistically significant at *P* < 0.05. Analyses were conducted using IBM SPSS Statistics version 27 for Windows.

### 2.4. Ethics approval

The study was conducted in accordance with the Declaration of Helsinki and approved by the Ethics Committee of Kanazawa University (protocol code No. 1491; date of approval, December 12, 2013). All the data used in this study were consented by the participants for use in research purposes, including mortality linkage.

## 3. Results

Participant characteristics by sex are presented in Table 1. The final analysis included 2849 residents of Shika town, comprising 1275 males (44.75%) and 1574 females (55.25%) with mean ages of 64.00 years (SD = 12.21) and 65.44 years (SD = 13.12), respectively. Significant sex differences were observed. Males had higher: mortality rate (12.40% vs 7.8%; *P* < 0.001), mean BMI (23.57 kg/m^2^ vs 22.57 kg/m^2^; *P* < 0.001), alcohol intake (6.68 vs 1.29; *P* < 0.001), current tobacco smoking (32.70% vs 6.50%; *P* < 0.001), high blood pressure (33.20% vs 29.50%; *P* = 0.035), and diabetes (13.20% vs 8.50%; *P* < 0.001). On the other hand, females had higher frequency of hyperlipidemia (11.50% vs 18.70%; *P* < 0.001), mean age (64.00 vs 65.44; *P* = 0.003), chronic headache (0.90% vs 2.00%; *P* = 0.015), and knee/foot pain (*P* < 0.001).

**Table 1:** Participant characteristics by sex.

Characteristics	1275 males	1574 females	*P*
	N (%)	N (%)	
Nonsurvivor	158 (12.40)	123 (7.80)	**<0.001**
Age, y, mean (SD)	64.00 (12.21)	65.44 (13.12)	**0.003**
BMI (kg/m^2^), mean (SD)	23.57 (3.06)	22.57 (3.44)	**<0.001**
Alcohol (density method), energy/g, mean (SD)	6.68 (8.17)	1.29 (3.75)	**<0.001**
Tobacco smoking (yes)	417 (32.70)	102 (6.50)	**<0.001**
High blood pressure (yes)	424 (33.20)	465 (29.50)	**0.035**
Diabetes (yes)	168 (13.20)	134 (8.50)	**<0.001**
Hyperlipidemia (yes)	147 (11.50)	294 (18.70)	**<0.001**
Chronic body pain (yes)			
Head	11 (0.90)	31 (2.00)	**0.015**
Neck/shoulder/elbow/hand	194 (15.20)	247 (15.70)	0.720
Upper back/lower back/hip	244 (19.10)	329 (20.90)	0.238
Knee/foot	158 (12.40)	317 (20.10)	**<0.001**

Statistical significance is indicated by *P* < 0.05.

BMI, body mass index; kg/m^2^, kilogram per meter square; SD, standard deviation.

As shown in Table 2, significant differences were observed between survivors and nonsurvivors. Mean age was significantly higher in nonsurvivors (62.17 vs 76.89; 63.99 vs 82.55; *P* < 0.001) for both males and females, respectively. Tobacco smoking rates were significantly higher in survivors than in the nonsurvivor group (males: 35.00% vs 16.50%, *P* < 0.001; females: 6.90% vs 1.60%, *P* = 0.023). Diabetes (7.90% vs 15.40%, *P* = 0.004) and high blood pressure (28.80% vs 38.20%, *P* = 0.028) rates were significantly higher in nonsurvivor females.

**Table 2:** Difference in mortality rates by sex.

Characteristics	Mortality					
	Males			Females		
	1117 survivors	158 nonsurvivors	*P*	1451 survivors	123 nonsurvivors	*P*
	N (%)	N (%)		N (%)	N (%)	
Age, y, mean (SD)	62.17 (11.48)	76.89 (9.11)	**<0.001**	63.99 (12.31)	82.55 (9.89)	**<0.001**
BMI (kg/m^2^), mean (SD)	23.7 (3.05)	22.64 (3.02)	**<0.001**	22.64 (3.41)	21.71 (3.59)	**0.004**
Alcohol (density method), energy/g mean, (SD)	6.99 (8.28)	4.43 (6.96)	**<0.001**	1.34 (3.74)	0.66 (3.82)	0.055
Tobacco smoking (yes)	391 (35.00)	26 (16.50)	**<0.001**	100 (6.90)	2 (1.60)	**0.023**
High blood pressure (yes)	365 (32.60)	59 (37.30)	0.241	418 (28.80)	47 (38.20)	**0.028**
Diabetes (yes)	144 (12.90)	24 (15.20)	0.422	115 (7.90)	19 (15.40)	**0.004**
Hyperlipidemia (yes)	138 (12.30)	9 (5.70)	**0.014**	284 (19.60)	10 (8.10)	**0.002**
Chronic body pain (yes)						
Head	11 (1.00)	0 (0.00)	0.210	25 (1.70)	6 (4.90)	**0.016**
Neck/shoulder/elbow/hand	179 (16.00)	15 (9.50)	**0.033**	223 (15.40)	24 (19.50)	0.225
Upper back/lower back/hip	198 (17.70)	46 (29.10)	**<0.001**	291 (20.10)	38 (30.90)	**0.005**
Knee/foot	126 (11.30)	32 (20.30)	**0.001**	276 (19.00)	41 (33.30)	**<0.001**

Statistical significance is indicated by *P* < 0.05.

BMI, body mass index; kg/m^2^, kilogram per meter square; SD, standard deviation.

Regarding CP, headache rates were significantly higher in nonsurvivor females (1.70% vs 4.90%, *P* = 0.016) but not in males. The rate of upper back/lower back/hip pain (17.70% vs 29.10%; *P* < 0.001), (20.10% vs 30.90%; *P* = 0.005) and knee/foot pain (11.30% vs 20.30%; *P* = 0.001), (19.00% vs 33.30%; *P* < 0.001) were significantly higher among nonsurvivors in both males and females, respectively. In contrast, neck/shoulder/elbow/hand pain rates were significantly lower in nonsurvivor males (16.00% vs 9.50%, *P* = 0.033).

As shown in Table 3, significant differences were observed between survivors and nonsurvivors, stratified by age.

**Table 3:** Characteristics of participants aged ≤74 years and ≥75 years by sex.

2161 participants ≤74 y	Mortality					
	Males			Females		
Characteristics	955 survivors	57 nonsurvivors	*P*	1127 survivors	22 nonsurvivors	*P*
	N (%)	N (%)		N (%)	N (%)	
Age, y, mean (SD)	59.24 (9.60)	67.26 (5.18)	**<0.001**	59.23 (9.39)	65.32 (7.05)	**0.003**
BMI (kg/m^2^), mean (SD)	23.84 (3.05)	22.87 (2.77)	**0.019**	22.58 (3.39)	22.71 (4.40)	0.861
Alcohol (density method), energy/g, mean (SD)	7.51 (8.50)	6.68 (8.85)	0.473	1.63 (4.11)	1.22 (5.67)	0.651
Tobacco smoking (yes)	365 (38.20)	16 (28.10)	0.126	97 (8.60)	0 (0.00)	0.150
High blood pressure (yes)	296 (31.00)	23 (40.40)	0.138	272 (24.10)	8 (36.40)	0.186
Diabetes (yes)	119 (12.40)	12 (21.10)	0.060	82 (7.30)	3 (13.60)	0.259
Hyperlipidemia (yes)	117 (12.20)	3 (5.30)	0.113	908 (80.60)	21 (95.50)	0.079
Chronic body pain (yes)						
Head	10 (1.00)	0 (0.00)	0.438	16 (1.40)	2 (9.10)	**0.004**
Neck/shoulder/elbow/hand	154 (16.10)	6 (10.50)	0.262	175 (15.50)	7 (31.80)	**0.038**
Upper back/lower back/hip	165 (17.30)	15 (26.30)	0.082	183 (16.20)	6 (27.30)	0.167
Knee/foot	94 (9.80)	8 (14.00)	0.306	160 (14.20)	4 (18.20)	0.597

688 participants ≥75 y	162 survivors	101 nonsurvivors	*P*	324 survivors	101 nonsurvivors	*P*
Age, y, mean (SD)	79.49 (3.90)	82.33 (5.72)	**<0.001**	80.54 (4.37)	86.31 (5.42)	**<0.001**
BMI (kg/m^2^), mean (SD)	22.91 (2.93)	22.51 (3.16)	0.299	22.83 (3.51)	21.49 (3.37)	**<0.001**
Alcohol (density method), energy/g, mean (SD)	3.89 (5.97)	3.17 (5.26)	0.316	0.34 (1.59)	0.54 (3.31)	0.408
Tobacco smoking (yes)	26 (16.00)	10 (9.90)	0.158	3 (0.90)	2 (2.00)	0.391
High blood pressure (yes)	69 (42.60)	36 (35.60)	0.263	146 (45.10)	39 (38.60)	0.254
Diabetes (yes)	21 (13.00)	6 (5.90)	0.068	65 (20.10)	9 (8.90)	**0.010**
Hyperlipidemia (yes)	25 (15.40)	12 (11.90)	0.421	33 (10.20)	16 (15.80)	0.120
Chronic body pain (yes)						
Head	1 (0.60)	0 (0.00)	0.429	9 (2.80)	4 (4.00)	0.547
Neck/shoulder/elbow/hand	25 (15.40)	9 (8.90)	0.125	48 (14.80)	17 (16.80)	0.623
Upper back/lower back/hip	33 (20.40)	31 (30.70)	0.058	108 (33.30)	32 (31.70)	0.758
Knee/foot	32 (19.80)	24 (23.80)	0.440	116 (35.80)	37 (36.60)	0.879

Statistical significance is indicated by *P* < 0.05.

BMI, body mass index; kg/m^2^, kilogram per meter square; SD, standard deviation.

### 3.1. Participants aged ≤74 years

Nonsurvivors were significantly older (males: 59.24 vs 67.26, *P* < 0.001; females: 59.23 vs 65.32, *P* = 0.003) and male survivors had higher BMI (23.84 kg/m^2^ vs 22.87 kg/m^2^, *P* = 0.019). Female nonsurvivors had higher rates of chronic headache (1.40% vs 9.10%, *P* = 0.004) and neck/shoulder/elbow/hand pain (15.50% vs 31.80%, *P* = 0.038).

### 3.2. Participants aged ≥75 years

Nonsurvivors were significantly older than survivors (79.49 vs 82.33; 80.54 vs 86.31; *P* < 0.001 for both sexes). Female survivors had higher BMI (22.83 kg/m^2^ vs 21.49 kg/m^2^, *P* < 0.001) and diabetes prevalence (20.10% vs 8.90%, *P* = 0.010). Upper back/lower back/hip pain showed a potentially significant difference in males (20.40% vs 30.70%, *P* = 0.058).

**Figure 2.**
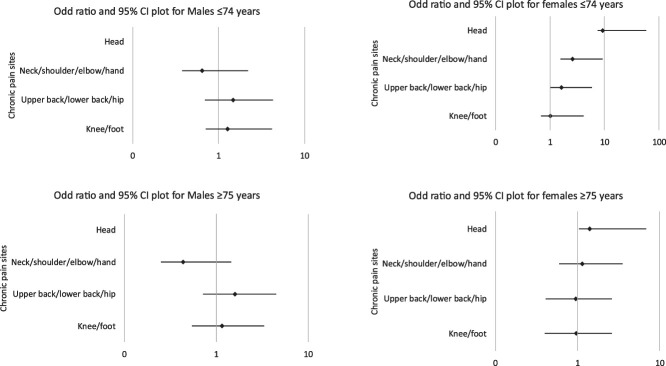
Showing the association between chronic body pain sites and mortality by age and sex.

Table 4 presents the results of a binary logistic regression analysis examining the relationship between CP and mortality among female participants (n = 1574), stratified by age groups (≤74 and ≥75 years), after adjusting for confounding factors. The analysis revealed that chronic headache among participants aged ≤74 years was independently associated with mortality (odds ratio [OR]: 9.238; 95% CI: 1.729, 49.352; *P* = 0.009). In addition, chronic neck/shoulder/elbow/hand pain was also significantly associated with mortality (OR: 2.586; 95% CI: 1.012, 6.608; *P* = 0.047) (Fig. 2). Among participants aged ≥75 years, no significant associations were found between chronic headache, neck/shoulder/elbow/hand pain, and mortality. Upper back/lower back/hip and foot/knee pain were not significantly associated with mortality in either age group.

**Table 4:** Binary logistic regression of mortality and chronic body pain.

Chronic body pain	Mortality			
	1149 females ≤74 y		425 females ≥75 y	
	95% CI for EXP (OR)	*P*	95% CI for EXP (OR)	*P*
	aOR (lower–upper)		aOR (lower–upper)	
Head	9.238 (1.729–49.352)	**0.009**	1.408 (0.365–5.438)	0.620
Neck/shoulder/elbow/hand	2.586 (1.012–6.608)	**0.047**	1.152 (0.554–2.399)	0.705
Upper back/lower back/hip	1.611 (0.610–4.259)	0.336	0.950 (0.541–1.668)	0.858
Knee/foot	1.012 (0.325–3.152)	0.984	0.962 (0.559–1.656)	0.888

Adjusted covariates (age, BMI, alcohol, smoking, hyperlipidemia, hypertension, and diabetes).

Statistical significance is indicated by *P* < 0.05.

aOR, adjusted odd ratio; BMI, body mass index; CI for EXP (OR), confidence interval for expected odd ratio; SD, standard deviation.

Table 5 presents the results of a binary logistic regression analysis examining the relationship between CP and mortality among male participants (n = 1275), stratified by age groups (≤74 and ≥75 years). The analysis revealed no statistically significant associations between CP and mortality in either age group (Fig. 2).

**Table 5:** Binary logistic regression of mortality and chronic body pain.

Chronic body pain	Mortality			
	1012 males ≤74 y		263 males ≥75 y	
	95% CI for EXP (OR)	*P*	95% CI for EXP (OR)	*P*
	aOR (lower–upper)		aOR (lower–upper)	
Head	0.000 (0.000–)	0.999	0.000 (0.000–)	1.000
Neck/shoulder/elbow/hand	0.642 (0.265–1.557)	0.327	0.433 (0.184–1.016)	0.054
Upper back/lower back/hip	1.470 (0.775–2.790)	0.238	1.600 (0.882–2.902)	0.122
Knee/foot	1.275 (0.564–2.882)	0.560	1.150 (0.607–2.180)	0.668

Adjusted covariates (age, BMI, alcohol, smoking, hyperlipidemia, hypertension, and diabetes).

Statistical significance is indicated by *P* < 0.05.

aOR, adjusted odd ratio; BMI, body mass index; CI for EXP (OR), confidence interval for expected odd ratio; SD, standard deviation.

## 4. Discussion

Our study of 2849 Shika town residents aged ≥40 years found that chronic headache and neck/shoulder/elbow/hand pain were independently associated with mortality in females ≤74 years old but not among males. Notably, these associations were not observed in males or in females ≥75 years old, suggesting age- and sex-specific relationships between CP and mortality. These findings are particularly concerning given that this age group is predominantly part of the active workforce.

The impact of chronic pain should not be underestimated, as it is a leading cause of disability, increased health care burden, loss of productivity, and increased comorbidities as captured in the ICD-11.^3^ Although CP has been explored in various studies, it is often considered a secondary factor or risk factor for other diseases, such as cardiovascular disease and mortality.^1,2,12,21,38,47,49^ Few studies have potentially examined the association between chronic regional pain and mortality, typically focusing on chronic widespread pain (CWP) or fibromyalgia.

Our study stands out by potentially linking CP to mortality after adjusting for confounding factors, and our findings align with those of several high-impact studies. Markedly, a cross-sectional study within the same cohort found high prevalence of chronic lumbar and neck/shoulder pain among working-aged individuals.^29^ Furthermore, a longitudinal study using NHANES data showed that chronic neck pain lasting over a year significantly increased osteoarthritis-related and all-cause mortalities,^8^ supporting our findings. Furthermore, a Finnish cohort study found that chronic pain increased mortality risk in the 18 to 49 age group for both sexes, with higher standardized mortality rates in women aged 60 to 69 years.^53^ Other authors found associations between lower baseline health-related quality of life, particularly psychosocial dimensions, to increased mortality risk among individuals with chronic pain.^21,28^ In addition, associations between CP and psychiatric disorders, such as depression and anxiety, have been reported among those with migraine or tension-type headache^28^; neck and shoulder pain.^21^ The mechanisms underlying these findings are likely complex, involving multiple systems, including the central nervous, autonomic, neuroendocrine, immune, vascular, and hematologic systems.^18^ Further research is needed to clarify these mechanisms, particularly by age and sex.

Our rural-based study found that women with chronic headache and neck/shoulder/elbow/hand pain were at higher risk of mortality, consistent with studies in the United States and Finland.^13,20,24^ These studies reported higher mortality rates associated with CP in older women in rural areas. Similarly, studies among Chinese adults and Taiwanese populations found that older individuals with CP had elevated mortality rates, with females experiencing higher rates of headaches, osteoporosis, and rheumatic arthritis.^6,46^ Our findings also align with NHANES studies, which showed that individuals with localized or widespread CP were at greater risk of all-cause mortality.^8,19,35^ The consistency of our results with these studies highlights the importance of addressing CP, particularly in middle-aged and older adult females in Japan.

These findings are consistent with a weekly report among inpatients showing a sixfold higher mortality rate among patients with severe CP compared to the general population.^48^ In addition, investigation among elderly Chinese individuals found associations between overall body pain, site-specific pain, and various chronic diseases.^22^ These studies further support the associations between CP and adverse health outcomes. Chronic pain has been associated to increased risk of cardiovascular events, including myocardial infarction, stroke, and heart failure, independent of traditional risk factors among participants of the UK Biobank.^12^ Moreover, systematic reviews have underscored the significant burden of CP on health care systems, socioeconomic status, productivity, and disability, as well as its functional, emotional, and social impacts.^4,15,37,49^ These findings emphasize the far-reaching consequences of CP in older adults. Without a doubt, these consequences are more severe in an aging population like this study participants. Further research is needed to clarify the direct link between chronic headache and neck/shoulder/elbow/hand pain and mortality, which could inform strategies to mitigate their impact on socioeconomic status, productivity, disability,^33^ and reduce the burden on health care.

This study's findings on the association between CP and mortality was supported by a 19-year Danish study and other studies.^19,25,28^ Unlike the Danish study, we adjusted for BMI^44^ as a covariate, providing a more robust analysis. The association between CP and mortality is consistent with existing literature^23^; although our study uniquely explored site-specific pain, adding new evident in literature regarding Japanese population. Thus, an analysis of 14,031 participants from NHANES revealed that the prevalence rates of rheumatoid arthritis and osteoporosis increase with age, adding that both osteoporosis and rheumatoid arthritis occur more frequently in women than in men, which is^27^ consistent with the findings of a systematic study which explored the prevalence of rheumatoid arthritis in 204 countries using 98 prevalence and 25 incidence studies.^5^ The above findings are all consistent with our results. Hence, osteoporosis and rheumatoid arthritis may have contributed to the strong association between CP and mortality in this study population.

Chronic pain is a prevalent condition in the general population, often comorbid with other ailments.^29^ However, CP can also be a standalone illness,^14^ and to better understand the relationship between pain and mortality, it is essential to examine site-specific pain rather than just CWP.^43^ Findings suggested that age-related changes in pain processing may contribute to diminished pain sensitivity in older adults,^45^ this could influence our findings among the older aged group. In addition, differences in pain reporting between men and women may play a role, because women tend to describe pain in more detail and with greater emotional sensitivity than men who use fewer words and focus on the sensory aspects of pain.

Chen et al.^6^ reported a higher prevalence of CP in women, with differing pain locations between sexes. The authors buttressed that men mostly suffer chronic headaches and shoulder and neck pain, whereas women suffer from chronic shoulder, stomach, abdomen, and waist pain.^6^ This study found no significant association between chronic headache and neck/shoulder/elbow/hand pain and mortality in men. In contrast, our results showed a strong and independent relationship between these pains and mortality in women aged ≤74 years. This study's findings are in contrast with the Italian study, which found an inverse association between neck pain and mortality in elderly individuals aged >65 years, potentially due to reduced sensitivity with age.^30^ Our study's focus on younger adults may explain the difference, as this group is less likely to experience age-related declines in pain sensitivity.

Our findings of a significant association between chronic pain and mortality in females align with the Japanese records in WHO database 2022, which reported higher mortality rates due to musculoskeletal issues in females (3258) than males (1650) aged ≥35 years.^53^ As one of the few studies to investigate the association between site-specific chronic pain and mortality in Japan, our research addresses growing concerns about the relationship between chronic pain and mortality.

This study's strengths include its high response rate and comprehensive coverage of the community, allowing for a robust analysis of the association between CP and mortality by sex and age over an 8.64-year follow-up period. However, the study's limitations include its inability to establish causation and the lack of data on cause of death, physical activity, mental health status, medication use, and pain duration or trajectory. More longitudinal studies with longer follow-up periods and more comprehensive data are needed to confirm causal relationships and further elucidate the complex dynamics between CP and mortality.

### 4.1. Implication of the study

Our findings can inform health care policy and practice, particularly in highlighting the need for health care workers to be vigilant about chronic headache and neck/shoulder/elbow/hand pain in women aged ≤74 years. By recognizing the association between these pains and mortality, health care providers can develop targeted interventions to improve outcomes for this population.

## 5. Conclusions

This study reveals an independent association between chronic headache and neck/shoulder/elbow/hand pain and mortality in women aged ≤74 years, even after adjusting for confounding factors. This association was not observed in men. There is no clear understanding of the implication of CP on mortality among women aged ≤74 years, although osteoporotic or rheumatoid diseases may play a moderating or modifying role. These findings underscore the significant impact of chronic pain on mortality in younger women.

## Disclosures

The authors declare no conflicts of interest. The funders had no role in the design of the study; in the collection, analyses, or interpretation of data; in the writing of the manuscript; or in the decision to publish the results.

## Supplemental digital content

Supplemental digital content associated with this article can be found online at http://links.lww.com/PR9/A358.

## Supplementary Material

SUPPLEMENTARY MATERIAL
