# Supplementary material for: Chronic pain and mortality risk among middle-age and older Japanese: the Shika town cohort study
Source: Pain Rep. 2025 Nov 12;10(6):e1361. doi: 10.1097/PR9.0000000000001361 (PMC12614683; doi:10.1097/PR9.0000000000001361)
Supplement: SUPPLEMENTARY MATERIAL [file painreports-10-e1361-s001.pdf]

Shika Town Health Promotion Questionnaire No. 2

Kanazawa University Faculty of Medicine, Department of Environmental Ecology and Public Health.

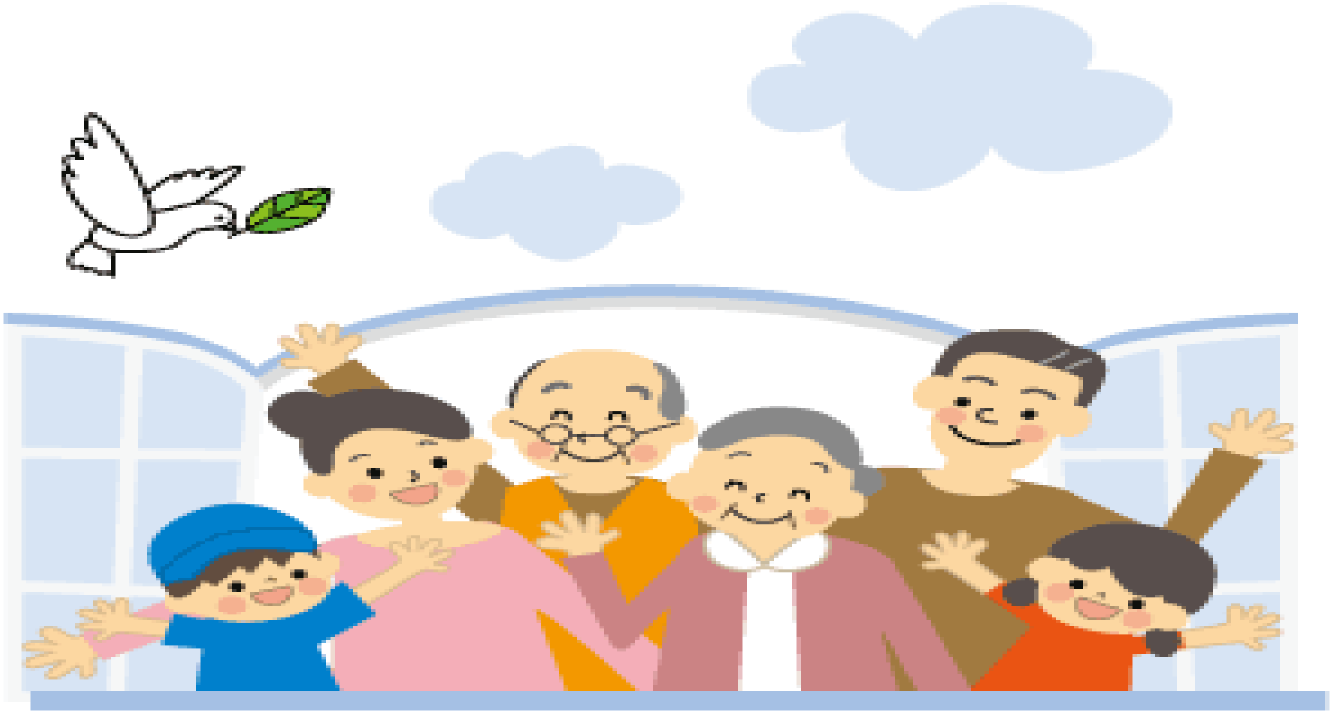

Shika Town Health Promotion Team:

Contact Information Telephone number 080-8697-1715 [Reception hours] Monday to Friday (excluding public holidays) 10:00 am to 4:00 pm

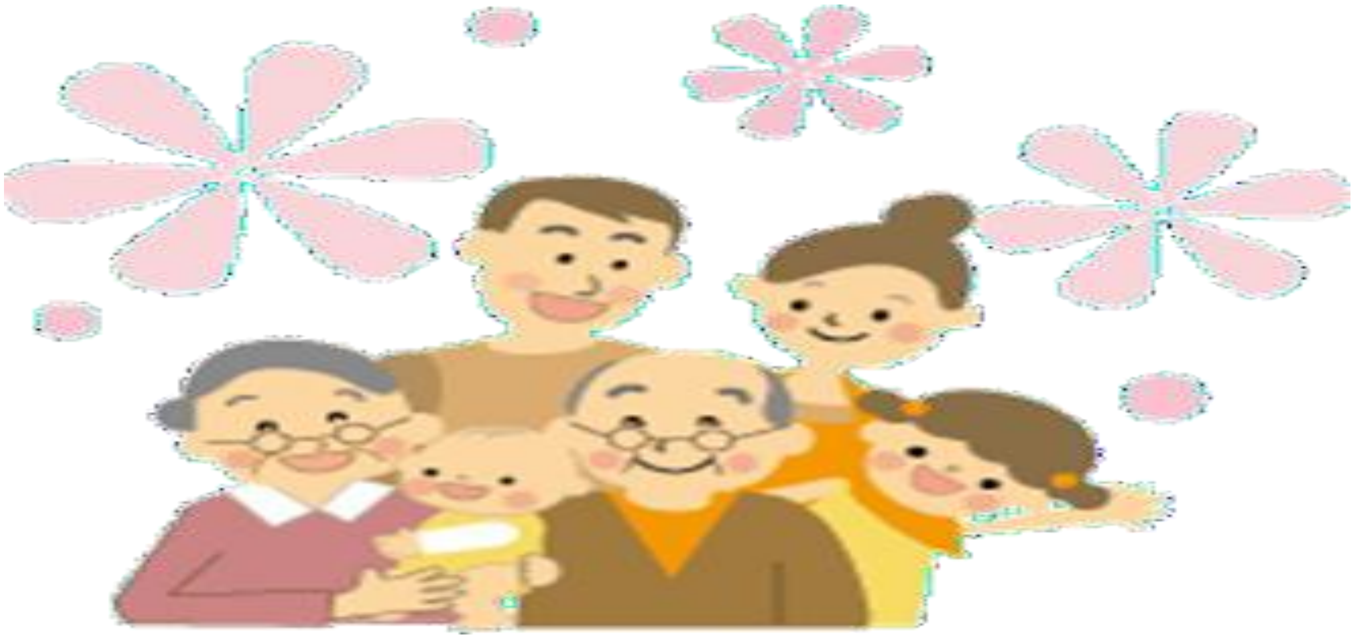

Shika Town Health Awareness Survey 2012 (this booklet)

Please fill out the survey questionnaire

Pain within three months or more.

* Please include pain that has persisted intermittently for more than three months but respond only if you have experienced pain within the past month and if the pain occurs at least twice a week.

Q1. Have you had pain for more than three months?

Please circle the one number that applies to you.

1. Yes → Please answer the questions below

2. No → End of question

* Regarding pain that has continued for more than three months , please answer from the most painful place to the third most painful place. If there is only one painful place, please answer only one, and if there are only two painful places, please answer only two.

Q2. Of the pain that has continued for more than three months , where is it most painful?

Please circle the one number that applies to you.

If you have pain on either side, please tell us which side hurts more.

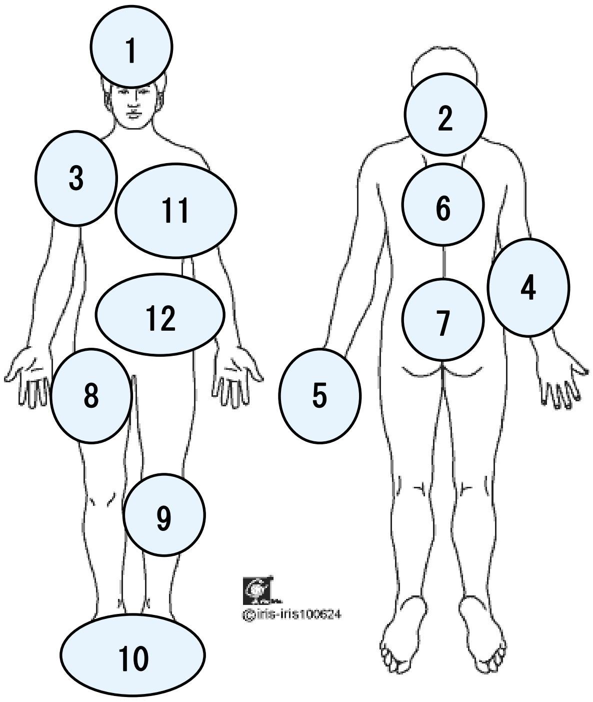

1 Head	2 Neck
3 Shoulder	4 Elbow
5 Hand	6 Upper back
7 Lower back	8 Hip
9 Knee	10 Foot
11 Chest	12 Stomach

Q3. How long has the pain been going on for?

Please circle the one number that applies to you.

1. 3 months to 6 months 2. 6 months to 1 year 3. 1 to 3 years 4. 3+ years

Q4. Please tell us how intense the pain is.

On a scale of 0 being no pain and 10 being the worst pain you can imagine , how bad is the pain?

Please circle the one number that applies to you.

Pain intensity

0 1 2 3 4 5 6 7 8 9 10

No pain the worst pain imaginable

Q5. Have you ever received treatment for the pain at a hospital, chiropractor, etc.?

Please circle the one number that applies to you.

1. Currently continuing treatment 2. Treatment has ended 3. I have never received

Please answer the questions below

End of question

Q6. How has the pain changed with treatment?

Please circle the one number that applies to you.

1. It got better 2. Somewhat improved 3. Does not change 4. Worse

Q7. Are you satisfied with the treatment?

Please circle the one number that applies to you.

1. Satisfaction 2. Somewhat satisfied 3. Somewhat dissatisfied 4. Dissatisfaction

If there is only one area of pain, you are done. If you have any other pain site, answer the questions below.

Q8. Of the pain that has continued for more than three months, where is the second most painful place?

Please circle the one number that applies to you .

If you have pain on either side, please tell us which side hurts more.

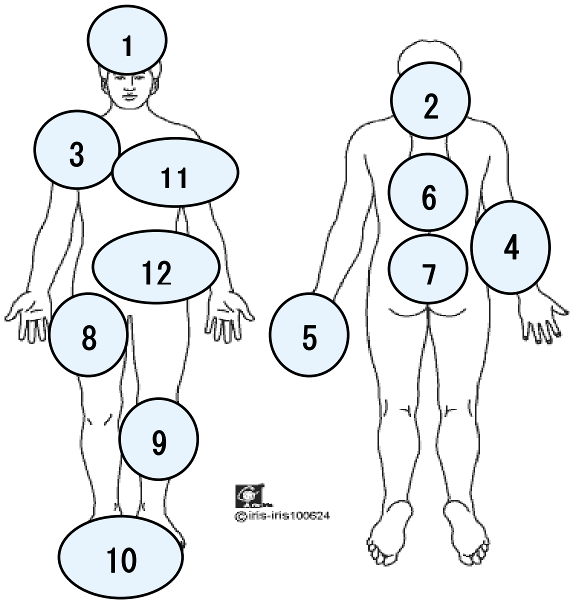

1 Head	2 Neck
3 Shoulder	4 Elbow
5 Hand	6 Upper back
7 Lower back	8 Hip
9 Knee	10 Foot
11 Chest	12 Stomach

Q9. How long has the pain lasted?

Please circle the one number that applies to you.

1. 3 months to 6 months 2. 6 months to 1 year 3. 1 to 3 years 4. 3+ years

Q10. Please tell us how intense the pain is.

On a scale of 0 being no pain and 10 being the worst pain you can imagine , how bad is the pain?

Please circle the one number that applies to you.

Pain intensity

0 1 2 3 4 5 6 7 8 9 10

No pain the worst pain imaginable

Q11. Have you ever received treatment for the pain at a hospital, chiropractor, etc.?

Please circle the one number that applies to you.

1. Currently continuing treatment 2. Treatment has ended 3. I have never received

Please answer the questions below

End of question

Q12. How has your pain changed with treatment?

Please circle the one number that applies to you.

1. It got better 2. Somewhat improved 3. Does not change 4. Worse

Q13. Are you satisfied with the treatment?

Please circle the one number that applies to you.

1. Satisfaction 2. Somewhat satisfied 3. Somewhat dissatisfied 4. Dissatisfaction

> If there are only two pain sites, you are done. If you have any other pain site, answer the questions below.

Q14. Of the pain that has continued for more than three months, where is the third most painful place?

Please circle the one number that applies to you .

If you have pain on either side, please tell us which side hurts more.

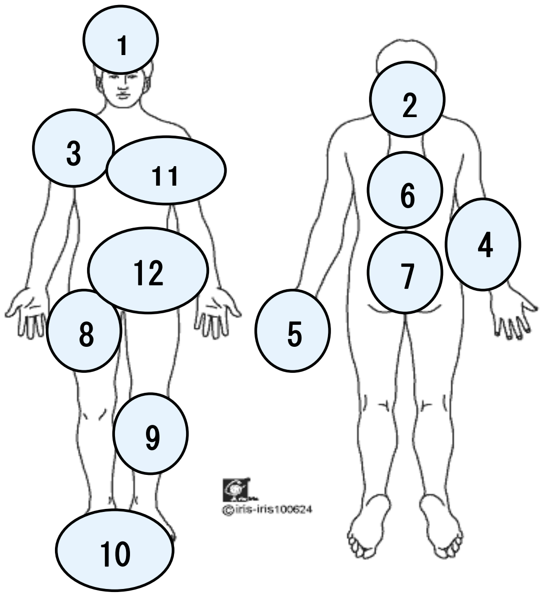

1 Head	2 Neck
3 Shoulder	4 Elbow
5 Hand	6 Upper back
7 Lower back	8 Hip
9 Knee	10 Foot
11 Chest	12 Stomach

Q15. How long has the pain lasted?

Please circle the one number that applies to you.

1. 3 months to 6 months 2. 6 months to 1 year 3. 1 to 3 years 4. 3+ years

Q16. Please tell us how intense the pain is.

On a scale of 0 being no pain and 10 being the worst pain you can imagine , how bad is the pain?

Please circle the one number that applies to you.

Pain intensity

0	1	2	3	4	5	6	7	8	9	10

No pain — the worst pain imaginable

Q17. Have you ever received treatment for the pain at a hospital, chiropractor, etc.?

Please circle the one number that applies to you.

1. Currently continuing treatment
2. Treatment has ended
3. I have never received

1, 2 → Please answer the questions below

3 → End of question

Q18. How has the pain changed with treatment?

Please circle the one number that applies to you.

1. It got better
2. Somewhat improved
3. Does not change
4. Worse

Q19. Are you satisfied with the treatment?

Please circle the one number that applies to you.

1. Satisfaction
2. Somewhat satisfied
3. Somewhat dissatisfied
4. Dissatisfaction
